# Less imageable words lead to more looks to blank locations during memory retrieval

**DOI:** 10.1007/s00426-018-1084-6

**Published:** 2018-09-01

**Authors:** Alper Kumcu, Robin L. Thompson

**Affiliations:** University of Birmingham School of Psychology, Birmingham, West Midlands UK

## Abstract

People revisit spatial locations of visually encoded information when they are asked to retrieve that information, even when the visual image is no longer present. Such “looking at nothing” during retrieval is likely modulated by memory load (i.e., mental effort to maintain and reconstruct information) and the strength of mental representations. We investigated whether words that are more difficult to remember also lead to more looks to relevant, blank locations. Participants were presented four nouns on a two by two grid. A number of lexico-semantic variables were controlled to form high-difficulty and low-difficulty noun sets. Results reveal more frequent looks to blank locations during retrieval of high-difficulty nouns compared to low-difficulty ones. Mixed-effects modelling demonstrates that imagery-related semantic factors (imageability and concreteness) predict looking at nothing during retrieval. Results provide the first direct evidence that looking at nothing is modulated by word difficulty and in particular, word imageability. Overall, the research provides substantial support to the integrated memory account for linguistic stimuli and looking at nothing as a form of mental imagery.

## Introduction

### Memory load and looking at nothing

Under grounded-embodied (Barsalou, 1999; Wilson, 2002) and extended (Clark & Chalmers, 1998) views of cognition, human memory exploits available sources in an opportunistic and efficient manner. This is particularly the case in the face of increased cognitive demands (Risko & Gilbert, 2016). Eye movements to “nothing” (i.e., blank locations in space) during memory retrieval is an example of exploitation of external sources to reduce memory load (i.e., the mental effort required for the maintenance and retrieval of information), and to increase memory efficiency (Johansson & Johansson, 2014; Scholz, Mehlhorn, & Krems, 2016). In looking at nothing, an integrated memory system attaches spatial information (represented as *spatial indices*) to information that needs to be retrieved during encoding (Ballard, Hayhoe, Pook, & Rao, 1997; Pylyshyn, 1989; Spivey, Richardson, & Fitneva, 2004). When the visual information itself is absent in retrieval, spatial indices trigger eye movements to the blank locations of the previously presented information (Ferreira, Apel, & Henderson, 2008; Richardson & Kirkham, 2004).

The present study aims to specify the conditions under which memory, as an internal faculty, relies on external support via eye movements. Looking at nothing, phenomenon presents an appropriate example of how eye movements are employed in such coordination between internal and so-called “external memory”. To this end, we examined the mechanisms of looking at nothing by investigating word retrieval with different lexico-semantic properties. To be more precise, we tested whether people rely more on the environmental support by looking at blank locations when remembering words that are more difficult to retrieve from memory due to their lexico-semantic properties.

Previously, eye closure, gaze aversion and other *nonvisual eye movements* that do not involve visual processing but accompany mental operations such as memory retrieval have been shown to be related to cognitive demands (see Salvi & Bowden, 2016 for a review). For instance, people disengage from environmental stimuli by shifting their gaze during challenging memory tasks to manage memory load (Doherty-Sneddon & Phelps, 2005; Glenberg, Schroeder, & Robertson, 1998). People also execute eye movements to search for nonvisual information stored in long-term memory and importantly, more frequent eye movements are executed when the task requires a more extensive and conceptually-driven memory search (i.e., verbal memory compared to visuospatial memory) (Ehrlichman & Micic, 2012; Ehrlichman & Barrett, 1983). Ballard et al. (1997) were among the first to suggest that the cognitive system can tap into eye movements at an embodied level to minimise memory load. Following a similar line of thought, Spivey and Geng (2000) speculated that people might not look at nothing when the answer in a memory task is salient enough to allow a response before any eye movements are produced. Johansson, Holsanova, Dewhurst, & Holmqvist (2012) also argued that eye movements could serve a supportive role during demanding tasks that involve visuospatial imagery. Similarly, Laeng, Bloem, D’Ascenzo and Tommasi (2014) suggest people attend blank locations only if additional spatial information could make a difference in memory retrieval. Based on these assumptions and the experimental evidence, a correlation can be expected between looks to “now-empty” locations and memory load, where higher load results in more frequent eye movements to blank locations.

Drawing on the potential trade-off between memory load and eye movements, two studies demonstrated that changing cognitive demands coming from task difficulty modulate looking at nothing. First, in Scholz et al. (2011), participants heard four sentences with a visual cue appearing for each sentence in one of the four quadrants across 12 trials. During a retrieval phase, participants’ recognition memory was probed with an auditory statement (correct vs. incorrect) querying a fact from one of the previously presented sentences. Sentences were repeated across the experiment and the proportion of fixations in the relevant, blank quadrant (where the corresponding cue had previously appeared) diminished after the first block as the retrieval task became easier through repetition. Memory load was high in the first block as the information to be retrieved was new and representations were weak. As a consequence, people looked at blank locations. However, the relevant information became familiar by the second and third blocks. Hence, internal memory no longer required an external aid. Consequently, memory load decreased and looking at nothing was not found.

Mental representations were also shown to play a fundamental role in the link between memory load and looking at nothing in a second study showing the effects of decreasing cognitive demands on looking at nothing. Wantz et al. (2015) presented participants with an object in one quadrant of a two by two grid. Memory for the presented objects was probed with a statement about one of the objects as participants looked at a blank screen. The retrieval phase was repeated across five sessions (immediately after the encoding, 5 min, 1 h, 24 h and 1 week after the encoding). More looks were directed towards the relevant, blank quadrant in the first three sessions compared to looks towards the irrelevant locations. However, looks towards the relevant quadrant were not greater in the latter two sessions, compared to looks towards other locations. In other words, people directed gaze less frequently to spatial locations associated with previously presented objects 1 day after the original encoding. This suggests that mental representations stabilise over time with repeated retrieval such that revisiting the original locations becomes unnecessary (see also Martarelli & Mast, 2013).

An integrated model of memory (Ferreira et al., 2008; Hoover & Richardson, 2008; Richardson, Altmann, Spivey, & Hoover, 2009; Spivey et al., 2004**)** accounts for the relationship between memory-guided eye movements and memory representations. Under this integrated model, mental representations (internal memory) and environmental sources internalised via eye movements (external memory) work cooperatively. According to the integrated model, representations are integrated and composed of spatial and linguistic input. If one part of the representation (e.g., linguistic) is reactivated through probing, other parts (e.g., spatial information) will be retrieved from memory as well. Task conditions such as repetition can make the linguistic component stronger, which stabilises the mental representations as a whole. In turn, people do not “need” to refer to spatial information for accurate retrieval. Thus, they look less at nothing. In other words, stronger internal memory and stronger mental representations require less environmental support through eye movements. In line with this view, Johansson, Holsanova, and Holmqvist (2011) showed that people with low-spatial imagery ability needed more eye movement support when describing a picture from memory using mental imagery. Kumcu and Thompson (2016) also reported less reliance on spatial indices during the retrieval of words among individuals with better visuospatial memory.

There is ample evidence showing that eye movements to blank locations are executed to offload memory work onto the environment during demanding memory tasks (see Risko & Gilbert, 2016 for a review). However, the nature of this behaviour remains elusive: when does the memory system “feel the necessity” to rely on environmental support and when does it turn back to internal memory? Does reliance on the environment change from item to item in a dynamic manner? If so, what type of information drives eye movements to blank locations? We addressed these questions in the current study. More precisely, we investigated whether words that are more difficult to remember also lead to more looks to relevant, blank locations. We hypothesise that fixations to blank locations are more likely to occur during retrieval of more difficult words compared to retrieval of easier words on two accounts: First, retrieval of difficult words impose a higher load on memory, which in turn, may make environmental support more appealing for the opportunistic memory system. Second, if the verbal component of the mental representation is weaker for more difficult words, people may rely on the spatial component more heavily by looking at the original location of the word to compensate for the verbal memory deficiency.

If the difficulty of individual items modulates eye movements, then we should expect both increases and decreases in looking percentages from trial to trial within the same session. Evidence for such eye movement behaviour would reveal the ability to switch between internal memory (representations) and so-called “external memory” (spatial indices via eye movements) in a flexible way. There is evidence for the effect of task difficulty on memory-guided eye movements as discussed above. However, we lack direct evidence that looking at nothing is modulated by word difficulty.

### Lexico-semantic variables and looking at nothing

Different word properties such as frequency have varying effects on how easily words are remembered. If words that are more difficult to remember lead to more reliance on environmental support via eye movements, the following question arises: which lexico-semantic variables contribute to looking at nothing? Word properties could affect memory-driven eye movements via two possible channels: memory load or mental imagery.

Individual properties of a word that make it difficult to remember (e.g., factors such as frequency and age of acquisition) increase memory load (e.g., Collette et al., 2001) and thus, might contribute to the tendency to look at nothing. In this regard, one prediction is that lexico-semantic variables modulate looking at nothing in proportion to their effects on memory performance. Distinctiveness enhances memorability in recognition (Eysenck & Eysenck, 1980; Schulman, 1967). In other words, people are less likely to detect previously seen words if they are not distinctive enough. According to a word difficulty prediction, therefore, variables which make a word less distinct and thus, more difficult to remember will also contribute to looks to blank locations during retrieval.

Many word properties play a role in verbal recognition memory through distinctiveness. For example, worse recognition performance has been evidenced for more frequent (Glanzer & Bowles, 1976), more available words (i.e., words that come to mind easily) (Rubin, 1983), early-acquired words (Dewhurst, Hitch, & Barry, 1998; but see Coltheart & Winograd, 1986) and words that have more orthographically similar neighbours (e.g., “book”—“hook”, “cook”, “crook”, etc.) (Cortese, Khanna, & Hacker, 2010; Cortese, McCarty, & Schock, 2015). Longer words are typically regarded as more distinct. However, more hits (i.e., correctly identifying previously seen words as old words) and fewer false alarms (i.e., identifying new words as previously seen) were reported for shorter words, suggesting that longer words tax the memory system by imposing more load (Cortese et al., 2010, 2015). In a typical recognition memory paradigm, words are presented visually and hence, encoded and retrieved in written form. As a result, phonological effects have not been demonstrated. For example, phonological similarity (i.e., having more neighbours that sound similar) does not appear to predict recognition memory accuracy as opposed to orthographic similarity (e.g., Cortese et al., 2010, 2015). Pronounceability was reported to have limited effect on recall (Rubin & Friendly, 1986) and its effect on recognition memory is not clear.

Imageability has a critical role in verbal memory (see Paivio, 1991; Schwanenflugel, 1991 for reviews). Imageability is defined as the extent to which a word evokes a mental image (Paivio, Yuille, & Madigan, 1968). For example, “apple” is a highly imageable word in that its meaning can quickly bring a salient picture to mind, which would be similar for most people. Whereas, the same cannot be said for low-imageable words such as “offer” or “coincidence”. Although these words can also stimulate images to a certain degree, they would not be as strong as those associated with high-imageable words. It is well established that words associated with perceptually salient, highly imageable objects/concepts are better remembered than those associated with low-imageable objects/concepts (see Marschark & Cornoldi, 1991 for a review). Imageability was shown to be one of the strongest predictors of recognition memory (e.g., Cortese et al., 2010, 2015) and recall (Rubin & Friendly, 1986) relative to other variables.

One prediction tested here is that imageability modulates looking at nothing due to its contribution to the mental image of the target word’s referent. Decades of evidence have demonstrated that eye movements are instrumental in mental imagery processes (see Mast & Kosslyn, 2002 for a review). For instance, Noton and Stark (1971) showed that eye movements during imagery are similar to the movements during perception (*scanpath theory*). Specifically, people simulate perception during imagery by re-enacting the eye movements that are executed during viewing (Altmann, 2004; Johansson et al., 2012; Laeng & Teodorescu, 2002; but see Johansson, Holsanova, & Holmqvist, 2006). Further, eye movements in mental imagery appear to support the image generation process (Johansson et al., 2012; Laeng et al., 2014). For instance, the degree of similarity in scanpaths between perception and imagery predicts the accuracy of memory for the visual scene (Laeng & Teodorescu, 2002).

Previously encoded visual or verbal information is “recreated” without any visual stimulus when people attend to blank locations during retrieval. Thus, looking at nothing involves visuospatial mental imagery by nature. Low-imageable words are expected to have weaker mental images as opposed to high-imageable words. In the face of weak mental images, people could rely more on external support by looking at blank locations to meet the imagery deficit. Alternatively, participants might treat the words as “picture-like” orthographic units when remembering them on a blank screen. In such a case, mental images are expected to reflect the physical, perceptual properties of the words (e.g., number and shape of letters) rather than conceptual elements activated by word meanings (see Hunt & Elliot, 1980). Under this prediction, orthographic properties, namely word length, number of syllables and orthographical similarity, would be expected to regulate fixations to the blank location.

In light of the research discussed above, we selected ten variables to control the words in the current study (imageability, concreteness^1^, context availability, pronounceability, age of acquisition, frequency, syllable length, length in letters, phonological and orthographic similarity). Mixed-effects models were fit to reveal the predictors of looking at nothing. It is important to note that imageability is a crucial predictor in both word difficulty and mental imagery predictions. Thus, models were fit for the variables predicting memory performance as well. If memory-guided eye movements are modulated mainly by word difficulty and memory load, predictors of memory performance should also predict looking at nothing. If mental imagery, in particular, modulates looks to blank locations, then imageability should stand out as a critical predictor of looking at nothing. If mental images corresponding to words are based on orthographic properties, word length (in letters and syllables) and orthographical similarity rather than imageability should play a role in eye movements to blank locations.

### Spatial interference between encoding and retrieval

Finally, we aimed to follow the experimental design in Kumcu and Thompson (2016) for consistency and comparison. Thus, participants were presented black dots as unrelated visual cues between encoding and retrieval phases. Cues were either congruent (shown in the same location as to the original location of the probe word) or incongruent (shown in a diagonal location as to the original location of the probe word) in addition to a “pure” looking at nothing condition without any cue. The cueing condition was designed to guide participants’ attention and eye movements to the location of the information held in memory (congruent cue) or away from it (incongruent cue) before retrieval.

There is evidence that additional visual processing within the looking at nothing paradigm has consequences both on memory performance and eye movements. For example, in Scholz, Klichowicz and Krems (2018), participants were asked to judge the truth of a sentence they had encoded in a grid location. At the same time, they were asked to attend a visual tracking task (Thomas & Lleras, 2009). In this task, random string of digits from 0 to 9 appeared on the screen and participants had to press a button whenever the digits appeared. Importantly, digits always appeared in the same location of the grid in a trial; that is, either congruent or incongruent locations with the location associated with the sentence to be retrieved. In one condition (overt attention), participants were asked to gaze freely as the visual tracking task occurred on the retrieval screen. In the other (covert attention), participants were asked to fixate on the centre and respond when the digits appeared. Participants were less accurate when the digit appeared in the incongruent locations compared to the congruent locations both under overt or covert attention conditions. Similarly, Kumcu and Thompson (2016) showed that a visual cue shown between the encoding and retrieval stages which is congruent with the location of the to-be-remembered word reinforces the spatial index of the word and thus, amplifies looking at nothing. On the other hand, an incongruent visual cue interferes with spatial indexing of the probe word and leads to the disruption of looks to relevant, blank locations.

In the present study, we investigated how spatial cues modulate the link between retrieval difficulty due to lexico-semantic variables and looking behaviour/memory performance. In particular, we tested how imageability affects looking at nothing in congruent and no cue conditions, respectively. If mental imagery is a reinstatement of previous perceptions in the absence of any stimulus (Hebb, 1968; Kosslyn, Thompson, & Ganis, 2006), the effect of imageability on looking at nothing should be stronger in a no cue condition in comparison with a congruent cue condition. There is overwhelming empirical evidence that actual visual perception and visual mental imagery share common mechanisms and influence each other (Cichy, Heinzle, & Haynes, 2012; Ganis, Thompson, & Kosslyn, 2004; Kosslyn, Thompson, & Alpert, 1997; Perky, 1910). Hence, visual perception of a cue could interfere with the generation of a mental image invoked with words under congruent cue condition. In turn, this could attenuate the effect of word imageability on looking at nothing. Whereas, word imageability could modulate looking behaviour under no cue condition; that is, when there is no visual information to interfere between encoding and retrieval phases.

## Method

### Participants

The experiment was carried out with 48 students at the University of Birmingham (nine males; *M*_age_ = 20.06, SD = 2.30, range 18–29). 75% of them were psychology students. All participants were monolingual native speakers of British English as determined with the Language History Questionnaire version 2.0 (Li, Zhang, Tsai, & Puls, 2013). Participants reported normal or corrected-to-normal vision, no speech or hearing difficulties and no history of any neurological disorder. They received either £10 (*n* = 27) or course credit (*n* = 21) for participation. All participants were fully informed about the details of the experimental procedure and gave written consent. Post-experiment debriefing revealed that all participants were naïve to the purpose of the experiment.

### Materials

There were 180 trials involving 810 unique nouns in total. All words were drawn from the extensions of Paivio, Yuille and Madigan norms for 925 nouns (Clark & Paivio, 2004). The word pool was filtered to exclude words shorter than 3 letters and longer than 11 letters.

Trials were evenly divided into two (*n* = 90) as high-difficulty and low-difficulty word groups based on the mean imageability of the whole set (4.99). It is not viable to manipulate one dimension by holding others constant due to intercorrelations between the variables. Thus, high-difficulty words were less imageable, more abstract, less available, less pronounceable, learnt later in life, longer (both in number of letters and syllables) and had less phonologic and orthographic similarity with other words in the language (see Table 1).

**Table 1:** Differences in lexico-semantic variables between high- and low-difficulty words shown as mean values, standard deviations in parentheses and Welch’s *t* test statistics

Variable	High-difficulty words	Low-difficulty words	*t*	*p*
Imageability	3.78 (0.88)	6.19 (0.43)	49.55	< 0.0001
Concreteness	3.55 (1.45)	6.51 (0.65)	37.41	< 0.0001
Length in letters	7.45 (1.88)	6.13 (1.78)	− 10.30	< 0.0001
Number of syllables	2.59 (0.90)	1.85 (0.77)	− 12.64	< 0.0001
Orthographic similarity	2.89 (0.52)	3.15 (0.73)	5.63	< 0.0001
Phonological similarity	2.80 (0.73)	3.32 (1.04)	8.22	< 0.0001
Pronounceability	6.23 (0.63)	6.53 (0.43)	8.12	< 0.0001
Age of acquisition	4.96 (0.89)	3.64 (1.05)	− 19.29	< 0.0001
Availability	2.07 (0.80)	2.28 (0.78)	3.68	< 0.001
Frequency	1.10 (0.70)	1.16 (0.66)	1.09	0.28

Both high- and low-difficulty groups were further divided into yes and no trials (*n* = 45). Probe words in the yes trials were among the four study words in the encoding phase, whereas a different, not seen, word was probed in the “no trials”. There were no significant differences in any of the variables between yes and no groups within high- and low-difficulty word groups (all *p*s > 0.05).

Words were then grouped into smaller trial sets of four (yes trials) and five words (no trials). Words within sets were matched on all variables (all SDs < 2.00) both in the yes and no trials. Words were further controlled such that no word started with the same letter or had any semantic relationship with any other word in the set. Monosyllabic, disyllabic and trisyllabic words were evenly distributed [e.g., (3, 3, 3, 3), (1, 2, 1, 2) or (3, 2, 3, 2, 3), etc.].

The word in each trial set with median imageability was selected as the probe leaving the others as distractors. Welch’s *t* tests revealed no significant differences between the probe and distractor words in any of the variables or in any of the four sub groups (i.e., high difficulty yes, low difficulty yes, high difficulty no, low difficulty no) (all *p*s > 0.05). Thus, any word among the four or five words in each trial set was as likely to be remembered as any other word in the same set.

Finally, we formed 180 unique mathematical equations [e.g., (2 × 3) − (2 + 3) = 1] to present as memory interference between encoding and retrieval phases (see Conway & Engle, 1996 for a similar design). Half of the equations were correct. Incorrect equations were further divided into two equal groups: The results were either plus or minus one of the correct result.

### Apparatus

Stimuli were presented on a TFT LCD 22-in. widescreen monitor operating at 60 Hz with a resolution of 1680 × 1050 pixels (501.7 mm × 337.4 mm). The monitor was placed 640 mm in front of the participant. A chin and forehead rest was used to reduce head movements. Participants’ eye movements were monitored using SR EyeLink 1000 (sampling rate: 1000 Hz, spatial resolution < 0.5°, http://sr-research.com/eyelink1000.html). Participants viewed the visual material with both eyes but only the left eye was tracked. Auditory material was produced by a native female speaker of British English in a sound attenuated room and recorded using Audacity (version 2.1.10, https://www.audacityteam.org). Participants responded (yes/no they had seen the word) by pressing one of two keys on a standard keyboard. Eye movement data were extracted using the SR EyeLink Data Viewer (version 2.4.0.198, https://www.sr-research.com/data-viewer/). No drift or blink correction procedure was applied.

Data were analysed and visualised in R programming language and environment (R Core Team, 2017). Mixed-effects models were constructed with *lme4* package (Bates, Mächler, Bolker, & Walker, 2015). Significant values of the coefficients in models were computed based on the t-distribution using the Satterthwaite approximation with *lmerTest* package (Kuznetsova, Brockhoff, & Christensen, 2015).

### Procedure

**Fig. 1:**
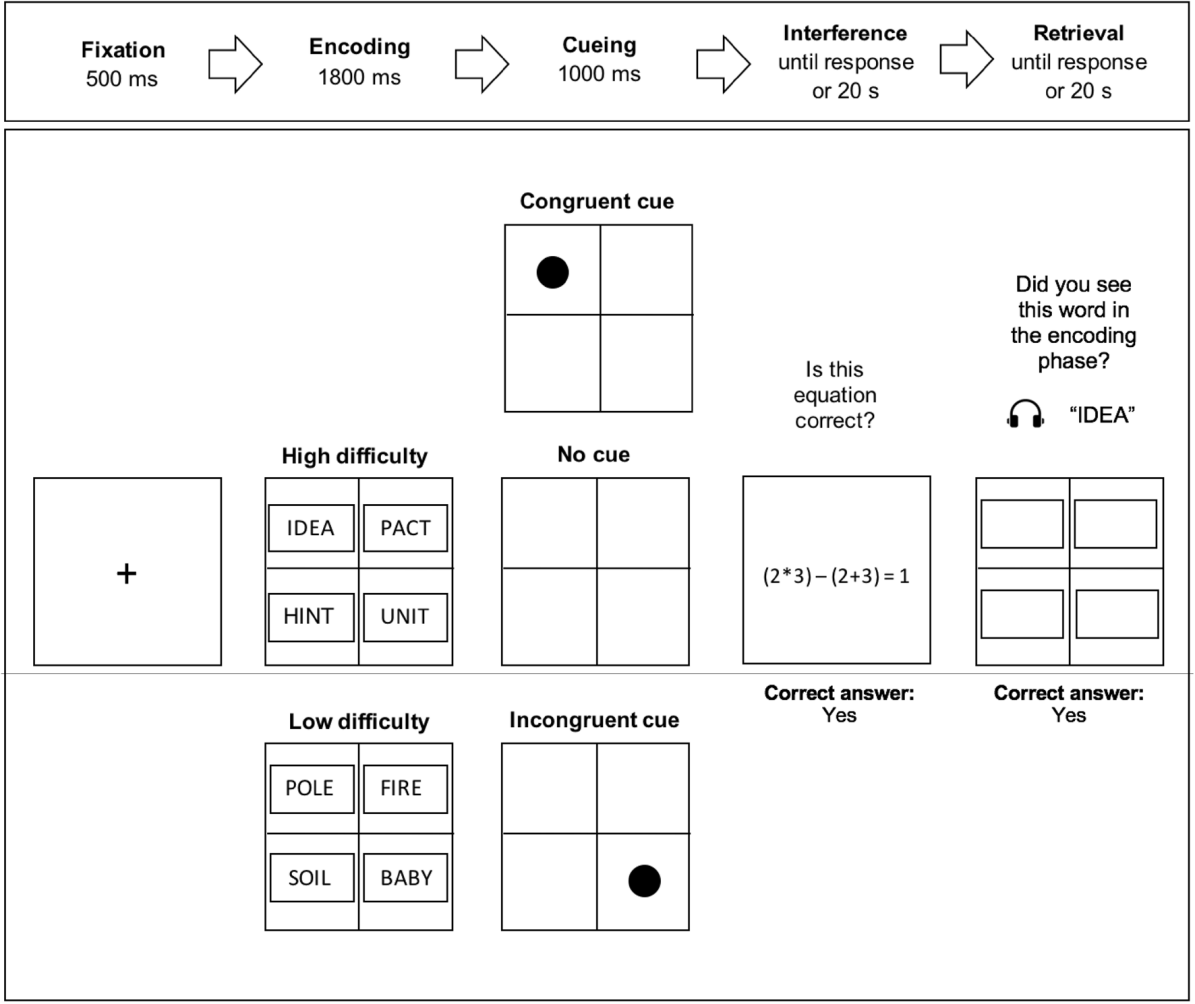
A schematic illustration of the temporal order of events in an example trial showing high- and low-difficulty word conditions and three different cue conditions. In this example, the relevant quadrant is the top left location, where the probe word (i.e., IDEA) appears

We followed the procedure in Kumcu and Thompson (2016). Eye tracking started with a standard nine-point calibration and validation, which confirmed high data quality (average calibration error < 1° and maximum calibration error < 1.50°). As spelled out in detail below, each trial was composed of five consecutive phases: (1) fixation, (2) encoding, (3) cueing, (4) interference and (5) retrieval (see Fig. 1). The task was to decide whether an auditorily presented word had appeared before or not (i.e., yes/no verbal recognition memory test). As soon as the participants made yes/no judgement by hitting one of the response buttons, the trial ended, and a new encoding phase began.

(1) Fixation: a fixation cross appeared at the centre of the screen for 500 ms. (2) Encoding: participants were presented four words in capital letters on a 2 × 2 grid for 1800 ms. Words (Times New Roman, font size = 40) were centrally placed in rectangular boxes (300 × 85 in pixels, 8° × 2.4° of visual angle). Word difficulty was a within-subject variable and all participants saw the high- and low-difficulty words. (3) Cueing: a flashing black dot appeared in cue trials for 1000 ms either in the same (congruent cue) or in the diagonal quadrant (incongruent cue) as the original location of the probe word in the encoding phase. There was also a third condition where no cue was presented between encoding and interference. Cue condition was a within-subject variable and three cue conditions were randomly presented in a session. That said, an equal number of random participants (*n* = 16) saw the same probe word with a congruent cue, an incongruent cue or without any cue. (4) Interference: participants were presented a mathematical equation [e.g., (2 × 3) − (2 + 3) = 1] and asked to identify whether the equation was correct or not within 20,000 ms (or they timed-out). (5) Retrieval: the probe word was auditorily presented as participants looked at the blank grid with empty boxes. There was a 500-ms gap between the presentation of the blank retrieval screen and the presentation of the probe word. Participants were asked to make an unspeeded yes/no judgement to determine whether they had seen the probe word among the four words shown in the encoding phase within 20,000 ms (or they timed-out).

The order of trials and equations were fully randomised independent of each other. The location of all words in all conditions was counterbalanced with Latin Square design to control gaze biases so that each word appeared an equal number of times in each location of the grid. The experiment was divided into four equal blocks with 45 trials in each block and there was a short pause between blocks. A typical session lasted approximately 100 min, including consent and setting up the eye tracker. Overall accuracy in interference equations and in the recognition memory test for words were 88% and 85%, respectively, suggesting that participants attended to the task with high concentration.

## Results

Results were analysed in two parts as memory performance and looking behaviour.

*Memory performance* Hit rate, hit latency and correct rejection rate were used as measures of memory performance. Hit rate was the proportion of yes trials to which the participants correctly responded yes. Correct rejection rate was the proportion of no trials to which the participants correctly responded no. Hit latency was the time in milliseconds between the onset of auditory presentation of the probe word and correct keyboard response. Participants were not instructed to make speeded response in the current paradigm. Nevertheless, hit latencies were reported to verify and complement the indicators of memory performance based on accuracy.

*Looking behaviour* Fixation percentage was used as the main gaze measure and dependent variable as in previous looking at nothing studies discussed above (e.g., Wantz et al., 2015). Fixation percentage (or fixation frequency) is the percentage of fixations in a trial falling within a particular interest area in proportion to total fixations in a trial. Thus, it was computed by dividing the number of fixations on each quadrant to the total number of fixations during the retrieval phase (see Wenzel, Golenia, & Blankertz, 2016 for a similar computation and use of fixation frequency).

Proportion of fixations was selected as the main indicator of looking behaviour on two grounds: first, it is immune to differences in durations. To be more precise, fixation percentage was considered appropriate particularly for comparing two different conditions (high- and low-difficulty words) with varying trial durations due to differences in word length between high- and low-difficulty words. Second, we assumed that fixations rather than the time spent on particular region (i.e., dwell time per quadrant) are important for the link between memory and eye movements. Fixation-based measures are reliable indicators of memory load and attention in a given location (e.g., Just & Carpenter, 1980; Meghanathan, van Leeuwen, & Nikolaev, 2015). As dwell time includes saccadic movements in addition to fixations, we preferred fixation percentage over dwell time percentage as a more refined indicator of looking at nothing. Accordingly, we expected that participants would fixate on the relevant quadrant to derive support from the environment.

Four rectangular interest areas corresponding to the quadrants were identified. All interest areas were of the same size (502 × 368 in pixels, 13.4° × 10.6° of visual angle). They framed the rectangular boxes that words were presented in (see Fig. 1) and were not contiguous. Proportion of fixations accrued on the interest areas during the retrieval phase (from the onset of auditory presentation of the probe word until the participant’s response) was calculated. Fixations were a minimum duration of 40 ms. First fixations and fixations outside the interest areas (8.62%) were omitted. Only hits (i.e., correct responses) in yes trials were included in the fixation analyses. Fixation percentages allocated to the three quadrants that did not contain the target probe word were averaged into one and analysed against the relevant quadrant in which the probe word was seen.

### Mixed-effects modelling

Data were analysed using linear and binomial logit mixed-effects modelling. Linear models were fit for continuous target variables (hit latency and fixation percentage). Binomial models were fit for categorical target variables (hit rate and correct rejection rate) and with *bobyqa* optimiser to prevent non-convergence. Participants and items were treated as random effects to explain by-participant and by-item variation (Baayen, Davidson, & Bates, 2008).

We started fitting models by building the random effects structure and followed a maximal approach. That is, random effects were included as both random intercepts and correlated random slopes (random variations) as long as they converged and were justified by the data (Barr, Levy, Scheepers, & Tily, 2013). Random intercepts and slopes were included even if they did not improve the model fit to control for possible dependence due to repeated measures or order effects.

Two approaches were adopted when building the fixed effects structure. Contribution of a fixed effect was investigated by comparing a full model containing the effect in question against a reduced model in which only that effect was removed, or a null model without any fixed effects (Winter, 2013). Best-fit model was specified by starting with a full model which included all fixed effects and their interactions (Bates et al., 2015). The full model was then reduced systematically in each step until the null model. Models were then compared using *anova* function to identify the model offering the best-fit by Akaike information criterion (AIC) and Bayesian information criterion (BIC), where lower is better in both cases (Hilbe, 2011) (see Appendix Tables 3, 4, 5, 6 for the outputs of the best-fit models).

### Factor analysis

The effect of lexico-semantic predictors on looking at nothing and memory performance was examined. Thus, we conceptualised fixation percentage in blank location, hit rate, hit latency and correct rejection rate as a function of length in letters, syllable length, orthographic similarity, phonological similarity, age of acquisition, frequency, availability, pronounceability, imageability and concreteness. Visual inspections of residual plots did not reveal any obvious deviations from homoskedasticity or linearity.

However, diagnostic tests indicated collinearity between the predictors as identified with the correlation matrix, variance inflation factors (VIF) (*M* = 3.60, range = 2.42–5.13) and *κ* (96.34). To address collinearity, we performed exploratory factor analysis and clustered the variables in components. Results of Bartlett’s test of sphericity, *χ*^2^(45) = 17011.77, *p* < 0.0001 and Kaiser–Meyer–Olkin measure of sampling adequacy (0.78) supported the existence of factors within the data. Hence, we proceeded to conduct the factor analysis using a principal component analysis extraction method with an orthogonal (varimax) rotation method. Both Kaiser’s criterion and the scree test criterion indicated the presence of three factors in our data. This conclusion was also supported by the percentage of variance criterion (Hair, Black, Babin, & Anderson, 2009), which suggests that all retained factors should account for at least 60% of the total variance. The three-factor solution in our analysis explained 78.24% of the variance in the data. Communalities of all predictors were above 0.70 (mean communality = 78.23; see Table 2) suggesting that all measures were adequately accounted for by the three-factor solution. As suggested by Hair et al. (2009), only factor loadings above 0.40 (or below − 0.40) were considered to meet the minimal level for interpretation of factor structure. Factor loadings did not have substantial loadings on other factors and they showed particularly clean clustering (except for age of acquisition). Further, the factors themselves had substantial loadings only for those variables, thus other variables did not load on these factors.

**Table 2:** Varimax rotated factor-loadings and communalities of the predictors

Predictors	Factor			
	Imagery	Lexical	Length and similarity	*h* ^2^
Imageability	**0.91**	0.17	0.25	0.93
Concreteness	**0.92**	− 0.04	0.18	0.89
Frequency	− 0.05	**0.91**	0.00	0.82
Availability	0.01	**0.81**	0.29	0.74
Pronounceability	0.20	**0.79**	0.33	0.76
Age of acquisition	− 0.48	− **0.61**	− 0.37	0.74
Phonological similarity	0.14	0.20	**0.89**	0.85
Syllable length	− 0.18	− 0.17	− **0.87**	0.82
Length in letters	− 0.20	− 0.13	**− 0.84**	0.77
Orthographic similarity	0.22	0.30	**0.73**	0.68
Factor statistics				
Eigen value	2.10	2.67	3.22	7.99
Variance (%)	21.03	26.73	32.16	79.93

Bolded numbers indicate the groupings. Eigenvalues and percentage of variance are after rotation. *h*^2^ = communality

Three factors were interpreted as follows based on the loadings (see Table 2): (1) imagery: imageability and concreteness. (2) Lexical: age of acquisition, frequency, availability and pronounceability. (3) Length and similarity: Length in letters, syllable length, orthographic similarity and phonological similarity. Age of acquisition seemed to contribute to all three factors to a certain degree. It was, therefore, considered within the lexical factor on theoretical grounds, its loading and in line with previous factor analyses (e.g., Clark & Paivio, 2004) VIF of the factors were below one and thus, below our threshold of two.

Regression scores calculated for the three factors were employed both as predictors and random slopes in the subsequent linear mixed-effects multiple regression models. Regression scores were additionally recalculated for each subset in each analysis. As expected, factor analyses extracted similar factor loadings and produced the same three-factor solutions.

### Memory performance

Hit rate, hit latency and correct rejection rate as measures of memory performance were analysed using mixed-effects modelling.

#### Hit rate

First, we analysed whether there was a difference in hit rate across congruent and incongruent cue conditions. The fixed effect was cue location with two levels (congruent and incongruent cue). Imagery and length and similarity factors were added as random slopes into participants. Imagery, lexical and length and similarity factors were added as random slopes into items. Cue location did not improve the model fit when compared against a null model; *χ*^2^(1) = 1.16, *p* = 0.28. In other words, participants retrieved the probe words in incongruent cue condition (mean hit rate = 79%) as accurately as congruent cue condition (mean hit rate = 77%). Cue location did not improve the model fit either when no cue condition (mean hit rate = 78%) was included; *χ*^2^(2) = 1.18, *p* = 0.55.

Second, we examined lexico-semantic variables modulating hit rate. As reported above, we did not find any differences in hit rate across cue conditions. Thus, mixed-effects models including all cue conditions were fit. All factors (i.e., imagery, lexical and length and similarity factors) were added as random slopes both into participants and items. Imagery factor improved the model fit significantly; *χ*^2^(1) = 13.20, *p* = 0.0003. Length and similarity factor contributed to the model with even higher magnitude; *χ*^2^(1) = 24.49, *p* < 0.0001. Whereas, lexical factor was not predictive of hit rate; *χ*^2^(1) = 0.02, *p* = 0.90. The best-fit model converged with length and similarity and imagery factors; *χ*^2^(1) = 13.19, *p* = 0.0003. This model was supported by a large AIC difference of 11.2 and a BIC difference of 4.8 compared to the next best model converged with length and similarity factor only. Participants were more accurate when retrieving high imageable and concrete; *β* = 0.18, *z* = 3.96, *p* < 0.0001 and shorter and less similar words; *β* = 0.24, *z* = 4.86, *p* < 0.0001.

Length and similarity included four different variables (phonological similarity, orthographic similarity, length in letters and syllable length) which might have contradicting effects on the memory performance. We, therefore, fitted simpler models to identify the individual effects of the variables on hit rate within length and similarity factor. To avoid collinearity, we selected length in letters from the word length set and orthographic similarity from the similarity set as fixed effects (VIF < 2). Models including word length and orthographic similarity as random slopes within items and word length within participants indicated that hit rate was predicted by word length; *χ*^2^(1) = 23.89, *p* < 0.0001 but not orthographic similarity; *χ*^2^(1) = 0.12, *p* = 0.73. Models with syllable length and phonological similarity did not change the results.

#### Hit latency

Linear mixed-effects models were fit to identify any difference in hit latency between cue conditions. Imagery and length and similarity factors were added as random slopes into participants. Imagery, lexical and length and similarity factors were added as random slopes into items. As in hit rate, likelihood tests indicated that there was no difference in hit latency between congruent (mean hit latency = 1978.22 ms) or incongruent (mean hit latency = 2057.67 ms) cue conditions; *χ*^2^(1) = 2.16, *p* = 0.14. Results did not change when no cue condition (mean hit latency = 2027.86 ms) was included; *χ*^2^(2) = 2.04, *p* = 0.36.

Next, we investigated the effect of lexico-semantic factors on hit latency. Imagery, lexical and length and similarity factors were added as random slopes into participants. Imagery and length and similarity factors were added as random slopes into items. All three factors, that is, lexical factor; *χ*^2^(1) = 4.62, *p* = 0.03, imagery factor; *χ*^2^(1) = 6.14, *p* = 0.01 and with a considerably higher magnitude, length and similarity factor; *χ*^2^(1) = 34.29, *p* < 0.0001 predicted hit latency. Thus, the best-fit model converged with all three factors as the fixed effects; *χ*^2^(1) = 4.34, *p* = 0.04. Participants were faster to retrieve high imageable and concrete; *β* = − 48.66, *t* = 2.38, *p* = 0.02 and shorter and less similar words; *β* = − 83.24, *t* = 4.73, *p* < 0.0001. They were slower to retrieve more frequent, more available, more pronounceable words which were learned earlier in life; *β* = 41.49, *t* = 2.13, *p* = 0.04.

As in hit rate, simpler models with word length and orthographic similarity demonstrated that hit latency was predicted by word length; *χ*^2^(1) = 8.57 *p* = 0.003 but not orthographic similarity; *χ*^2^(1) = 0.49, *p* = 0.49. Models with syllable length and phonological similarity did not change the results. Correlation between variables within lexical factor (i.e., frequency, availability, pronounceability and age of acquisition) did not allow us to investigate their individual effects on hit latency due to high collinearity (VIF > 2).

#### Correct rejection rate

Correct rejection rate was the proportion of “no trials” to which the participants correctly responded no. Visual cues in yes trials were located according to the location of the probe words at encoding; whereas, a different, not seen, word was probed in no trials. Thus, “no trials” were not presented with different cue conditions and there is necessarily no effect of cue for these trials. As a result, only the effect of lexico-semantic variables on correct rejections was investigated.

Imagery, lexical and length and similarity factors were added as random slopes into items. Participants were added as a random intercept as the random-slope model did not converge. None of the lexico-semantic variables predicted correct rejection rate: [length and similarity factor; *χ*^2^(1) = 3.23, *p* = 0.07, imagery factor; *χ*^2^(1) = 0.13, *p* = 0.72, lexical factor; *χ*^2^(1) = 0.23, *p* = 0.63]. Word length and orthographic similarity as raw values rather than regression scores of the length and similarity factor did not predict correct rejection rate either.

### Looking behaviour

#### Looking at nothing

We first analysed whether participants looked at nothing during memory retrieval. In other words, we investigated whether there were more looks to relevant, blank locations where probe words were shown at the encoding stage compared to irrelevant, blank locations.

The target variable was fixation percentage in correctly answered yes trials. The fixed effect was quadrant with two levels (relevant and irrelevant quadrant). Imagery, lexical and length and similarity factors were added as random slopes into participants and items.

Quadrant improved the model fit when all cue conditions were factored in; *χ*^2^(1) = 8.60, *p* = 0.003. Models with a single cue condition indicated that quadrant improved the model fit in congruent; *χ*^2^(1) = 5.70, *p* = 0.02 and in no cue conditions; *χ*^2^(1) = 5.39, *p* = 0.02 but not in incongruent cue condition; *χ*^2^(1) = 0.13, *p* = 0.72. That is, participants looked more at the relevant location in congruent; *β* = 0.02, *t* = 2.39, *p* = 0.02 and no cue conditions; *β* = 0.02, *t* = 2.32, *p* = 0.02. However, they did not look at nothing when they were shown a visual cue that was incongruent with the original location of the probe word during encoding. Hence, we analysed the effect of word difficulty and lexico-semantic predictors on looking at nothing in congruent and no cue conditions.

#### Effect of word difficulty on looking at nothing

A further sub-analysis was performed on the two conditions with overall evidence of looking at nothing behaviour (congruent and no cue conditions), to determine the role of word difficulty. Linear mixed-effects models with high- and low-difficulty word sets were fit separately. The target variable was fixation percentage in correctly answered yes trials. Fixed effect was quadrant with two levels (relevant and irrelevant quadrant). All lexico-semantic factors were included into participants and items as random slopes.

**Fig. 2:**
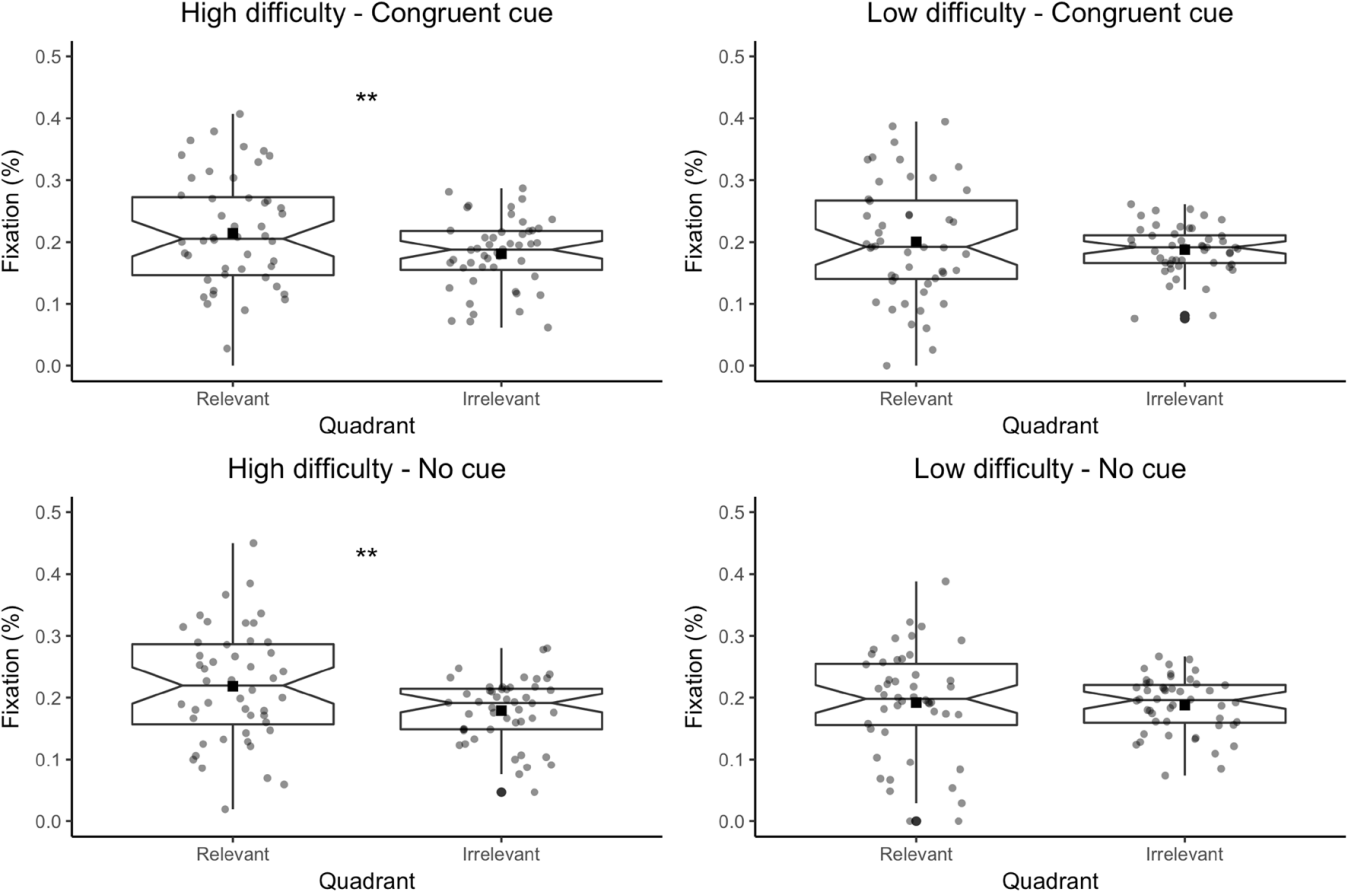
Percentages of fixation across relevant and irrelevant quadrants as participants retrieved high-difficulty and low-difficulty words in congruent and no cue conditions. Notched box plots show median (horizontal line), mean (black square), 95% confidence interval of the median (notch), interquartile range (the box), the first and the third quartiles (lower and upper ends of the box) and ranges (vertical line). Grey dots represent data points. **p ≤ 0.01

*Congruent cue condition* Quadrant improved the model fit for high difficulty; *χ*^2^(1) = 7.00, *p* = 0.008 but not low difficulty word set; *χ*^2^(1) = 0.59, *p* = 0.44. Participants looked more at the relevant quadrant than the irrelevant quadrant only when retrieving more difficult words; *β* = 0.04, *t* = 2.65, *p* = 0.008 (see Fig. 2).

*No cue condition* Quadrant improved the model fit in high-difficulty word set with a higher magnitude than the congruent cue condition; *χ*^2^(1) = 9.59, *p* = 0.002. Quadrant did not improve the model fit in low-difficulty word set; *χ*^2^(1) = 0.08, *p* = 0.77. Participants looked more at the relevant quadrant than the irrelevant quadrant when retrieving more difficult words; *β* = 0.04, *t* = 3.10, *p* = 0.002 (see Fig. 2).

Hit rate, hit latency and correct rejection rate between high- and low-difficulty words were compared with the aim of confirming the reliability of the difficulty manipulation. As expected, participants were more accurate; *β* = 0.36, *z* = 4.51, *p* < 0.0001 and faster; *β* = − 118.58 *t* = 3.37, *p* = 0.0008 when retrieving the low difficulty words. Further, low-difficulty words were subjected to less false alarms; *β* = 0.32, *z* = 2.62, *p* = 0.009.

#### Lexico-semantic predictors of looking at nothing

Next, we aimed to disambiguate the variables composing word difficulty. Effect of lexico-semantic predictors on looking at nothing behaviour was investigated. The target variable was fixation percentage in the relevant quadrant in correctly answered yes trials. Imagery and length and similarity factor were added as random slopes into participants. Imagery factor was added as random slopes into items.

*Congruent cue condition* Imagery factor; *χ*^2^(1) = 3.21, *p* = 0.07, length and similarity factor; *χ*^2^(1) = 1.27, *p* = 0.26 or lexical factor; *χ*^2^(1) = 0.24, *p* = 0.62 did not significantly predict fixation percentage in the relevant quadrant. Along with that, the best-fit model explaining the data was fit with imagery factor; *β* = − 0.02, *t* = 1.76, *p* = 0.08 based on AIC (0.74) and BIC differences (5.75) as to the next best model converged with imagery and length and similarity factors.

*No cue condition* Imagery factor predicted fixation percentage in the relevant quadrant; *χ*^2^(1) = 4.31, *p* = 0.04. Length and similarity; *χ*^2^(1) = 1.37, *p* = 0.24 or lexical factor; *χ*^2^(1) = 0.69, *p* = 0.41 were not significant predictors of looking at nothing. As a result, the best-fit model explains the data were the one with imagery as the fixed effect; *χ*^2^(1) = 4.06, *p* = 0.04. Higher imageability and concreteness predicted less fixations in the relevant quadrant; *β* = − 0.02, *t* = 2.03, *p* = 0.04.

#### Functionality of looking at nothing

The current experiment was not designed to test the functionality of looking behaviour in memory. Nevertheless, we examined whether memory performance was predicted by the proportion of fixations in the relevant, blank locations. Imagery factor was added as random slopes into participants. Imagery and length and similarity factors were added as random slopes into items. Looks to relevant, blank locations did not predict hit rate (congruent cue; *β* = 0.30, *z* = 1.24, *p* = 0.22, no cue; *β* = 0.02, *z* = 0.09, *p* = 0.93) or hit latency (congruent cue; *β* = − 64.77, *t* = 0.70, *p* = 0.49, no cue; *β* = 85.31, *t* = 0.78, *p* = 0.44). Models fit with high-difficulty or low-difficulty words only did not change the results.

## Discussion

We investigated (1) whether looking at nothing increases as participants are asked to study and retrieve more difficult words in a yes/no recognition memory paradigm and if so, (2) which lexico-semantic variable(s) predict the change in memory-guided eye movements to relevant, blank location. We further tested how a visual cue presented between encoding and retrieval stages modulates the effects of word difficulty and word properties on memory performance and looking at nothing.

As shown previously (Kumcu & Thompson, 2016), participants displayed looking at nothing behaviour in congruent and no cue conditions but not in the incongruent cue condition. Incongruent cues functioned as interference. That is, the spatial index associated with the probe word was updated with a visual cue that did not match with the word’s original location. In turn, the spatial index attached to the word and the spatial index attached to the visual cue competed and disrupted eye movements to blank locations. As looking at nothing behaviour was not exhibited in the incongruent cue condition, we investigated the effect of word difficulty and word properties on eye movements under congruent and no cue conditions.

We also examined memory performance under different retrieval conditions (cue conditions and high-difficulty vs. low-difficulty words) to verify the experimental manipulations and to compare against looking behaviour results. Unlike their effect on fixations, cue locations did not affect memory performance. Participants performed equally well under all retrieval conditions as demonstrated by both hit rate and hit latency. On the other hand, memory performance was superior in the retrieval of low-difficulty words in comparison with high-difficulty words as expected. Taken together, it is probable that visual cues were salient enough to modulate eye movements but not memory performance as memory performance was based more on the nonvisual, verbal parameters.

In line with our hypothesis, participants looked more at the blank, relevant quadrant compared to irrelevant quadrants when retrieving high-difficulty words but not low-difficulty words in congruent and no cue conditions. That is, participants relied on additional external sources when memory load was high, and they returned back to internal memory sources only when memory load decreased. We conceptualised memory load as the mental effort to maintain and reconstruct information as a function of difficulty. In this respect, such retrieval behaviour is in line with previous studies showing a proportional relation between memory load (via difficulty) and looking at nothing (Scholz et al., 2011; Wantz et al., 2015), where increase in load triggers eye movements. Additionally, the current study provides the first evidence that word difficulty as a function of lexico-semantic properties, modulates looking behaviour directly in memory.

Participants were likely to form stronger representations for low-difficulty words. Retrieving these words required less mental effort as opposed to words that are more difficult to remember. Consequently, internal memory involving mental representations was sufficient to retrieve the probe word and solve the memory problem (i.e., yes/no judgement) accurately. However, integrated memory engaged spatial indices when retrieving difficult words. Thereby, the verbal component of the memory representations corresponding to high-difficulty words was reinforced with stronger spatial information through eye movements and, as a consequence, memory load was alleviated.

High-difficulty words in the current study were in fact more distinctive than low-difficulty words in orthographic and phonological similarity, age of acquisition, availability and pronounceability. That is, high-difficulty words were less similar with others in the lexicon, learnt at later ages, less available (i.e., do not come to mind easily) and less pronounceable. If high-difficulty words were more distinctive in these variables, then why did participants still retrieve low-difficulty words more accurately than high-difficulty words? The variables which made high-difficulty words more distinctive (i.e., orthographic and phonological similarity, age of acquisition, availability and pronounceability) did not play a role in hit rate in the current study. Major predictors of hit rate, and by extension we assume, memory load, were word length, imageability and concreteness. Low-difficulty words were more imageable, more concrete and shorter (in letters and syllables). As a result, low-difficulty words imposed less load on memory and were retrieved more accurately.

The results have important implications for theories postulating that cognitive work can be offloaded onto the environment (Risko & Gilbert, 2016) and for the dynamics of looking at nothing, in particular. First, we clearly showed that participants switched from internal to external sources swiftly and efficiently from trial to trial given that difficulty was a randomised, within-subject variable in the current study. Such behaviour suggests that memory retrieval from internal and so-called “external memory” is not binary, but a dynamic process. Depending on the immediate memory load and strength of memory representations, retrieval behaviour hovers between the two extremes of a spectrum, where internal memory (representations) is on one end and external memory (spatial indices internalised via eye movements) on the other. Our results are in contrast to the accounts that reject the existence of mental representations (Chemero, 2011**)**. Rather, findings support the position that the external world has a supportive role in memory and environmental indices complement mental representations (Ferreira et al., 2008; Johansson et al., 2011; Richardson et al., 2009**)**. Eye movements are of particular importance in this pattern as they bind the internal representations to external information.

Whether or not eye movements are employed consciously as a memory strategy remains an unanswered question. There is evidence that explicit external support such as writing down to-be-remembered information is co-opted intentionally when it is offered as a choice (Risko & Dunn, 2015). We did not address this question in the current study. However, informal queries with the participants following the experiment revealed that they were not aware of the manipulation and did not look at the blank location with the intention of alleviating memory load (see also Johansson & Johansson, 2014; Scholz et al., 2016).

We did not present any evidence that looking at nothing has a functional role in memory. However, a number of studies demonstrated that looking at relevant, blank locations improves memory performance for both verbal and visual information (Johansson & Johansson, 2014; Scholz et al., 2018, 2016). What lies behind this discrepancy? We argue that the main reason is the difference in the experimental paradigms. Participants were instructed to look at either relevant or irrelevant quadrants during retrieval in the abovementioned studies evidencing functionality of looking at nothing. We did not design the current paradigm to test whether looking at nothing improves memory performance. Thus, eye movements were not manipulated, and all participants gazed freely during the retrieval phase. As a supplementary analysis, we tested whether fixations in the relevant, blank quadrant predict memory performance using mixed-effects models. As Martarelli, Chiquet, Laeng and Mast (2016) assert, the best way to understand functionality of looks to blank locations seems to be the manipulation of eye position at retrieval. In the current study, participants might have used other strategies to retrieve information from memory when they were not forced to look at certain positions on the screen.

Another possibility could be the difference in verbal information to be retrieved. In Scholz et al. (2011, 2016, 2018), participants encoded longer verbal information (i.e., factual sentences) and in the retrieval phase, a true/false statement probed participants’ memory. In our study, however, participants were asked to encode four single nouns shown simultaneously and memory was probed with another single noun. In line with our findings showing a link between word difficulty and looking at nothing, we speculate that functional role of eye movements in memory might emerge when memory load reaches a certain threshold (see Johansson et al., 2012; Laeng et al., 2014; Spivey & Geng, 2000). It is important to highlight that although participants looked more at relevant, blank locations than irrelevant, blank locations when remembering difficult words, looking at nothing did not predict memory performance in the retrieval of difficult words either. It is possible that maintaining and retrieving single words instead of longer verbal information were demanding enough to elicit looking at nothing behaviour but not demanding enough to allow for functional eye movements. Given the previous evidence discussed above, it is highly probable that the role played by eye movements in memory is beyond an epiphenomenal by-product of the retrieval mechanism (see also Hannula et al., 2010). That said, future studies should test the functional role of eye movements in memory under different retrieval conditions and with different verbal or visual material to systematise the effect.

Last, we used the term “eye movements” to describe the looking behaviour in the present study, following the practice in the literature (e.g., Johansson & Johansson, 2014; Richardson et al., 2009; Spivey & Geng, 2000). That said, it should be noted that we did not present any evidence that eye movements, in the strictest sense, are relevant in looking at nothing for visually presented single words as opposed to looking at nothing during mental imagery (e.g., Brandt & Stark, 1997).

Which word properties contribute to looking at nothing? Within the scope of the second research question, we explored lexico-semantic variables predicting eye movements to blank locations by clustering the variables into three factors (imagery, lexical and length and similarity factors). Use of word properties as independent and continuous variables instead of within difficulty categories in mixed-effects models eliminated the possibility of any confounding influence of stimuli design on the results. Imagery, that is, imageability and concreteness, was predictive of looking at blank locations during retrieval. Participants looked more at nothing when retrieving less imageable and more abstract words in no cue, that is, a “pure” looking at nothing condition. The effect of imageability and concreteness on fixations in the relevant quadrant under congruent cue condition was at a *p* level of 0.08. For the sake of simplicity, we will use the term “imageability” to refer both imageability and concreteness below.

Why did participants look more frequently to blank regions when retrieving less imageable words? As discussed in the introduction, imageability might have modulated eye movements in two different ways: due to its contribution to (1) word difficulty and thus, memory load or (2) mental imagery of words.

There is robust evidence that imageability is among the strongest predictors of performance in verbal recognition memory (Paivio, 1991). Accordingly, imageability might have affected fixations as the main moderator of word difficulty and memory load. However, retrieval performance was also predicted by length and similarity factor (length in letters, syllable length, phonological similarity and orthographic similarity) although it did not predict looking at nothing. This suggests that length and similarity (length, in particular) increased memory load as well. If the first account, that is, a difficulty/memory load account was indeed the only explanation for the effect of imageability on eye movements, length and similarity factor should have predicted eye movements as well.

As this was not the case, it appears more probable that imageability predicted looking at nothing mainly due to its contribution to the mental imagery of the words. We assume that participants relied more on mental imagery by looking at blank locations when the internal images activated by words fell short. This interpretation is also supported by the difference between congruent cue and no cue conditions in the effect of imageability on looking at nothing. The effect of imageability was revealed in the no cue condition but not in the congruent cue condition. As we discussed in the introduction, visual information emphasising the location of the probe word could have interfered with the mental imagery process and minimised the effect of imageability on looking at nothing.

In a nutshell, weaker mental images corresponding to less imageable words were compensated for by looking more at nothing during retrieval. The effect of imageability on eye movements also disproves the prediction that participants treated the words as orthographic units when remembering them on a blank screen. It is safe to assume that participants formed and relied on conceptual mental images rather than images reflecting the physical properties of words. The role of mental imagery in the current study is noteworthy considering that the participants were not instructed to generate mental images to retrieve the words (cf., Laeng & Teodorescu, 2002). Such a retrieval behaviour supports grounded-embodied and perceptual approaches to memory suggesting that retrieval is, in essence, imagining and simulating the encoding (Albers, Kok, Toni, Dijkerman, & De Lange, 2013; Glenberg, 1997; Jonides, Lacey, & Nee, 2005; Kent & Lamberts, 2008; Schacter et al., 2012; Wheeler, Petersen, & Buckner, 2000).

From a wider perspective, reducing internal demands is a crucial and consistent function of behaviours in which cognitive work is externalised (Gilbert, 2015; Melinger & Kita, 2007; Schönpflug, 1986). Here, we analysed language-based factors which influence the propensity to engage the external world with eye movements to minimise memory load. Our findings show that people rely more on spatial indices when retrieving low-imageable words. These findings can be considered compelling evidence for a flexible coordination of internal and external memory systems. Future studies should examine the temporal dynamics of the coordination between internal sources and external support.

## Appendix: Outputs of the best-fit mixed-effects models

### Looking behaviour

See Tables 3 and 4.

**Table 3:** Results of the mixed-effects model for fixation percentage in the relevant, blank quadrant under congruent cue condition offering the best fit by maximum likelihood

Fixed effects	*β*	SE (*β*)	*df*	*t*	*p*
(Intercept)	0.21	0.01	57.75	19.23	< 0.0001
Imagery	− 0.02	0.01	138.23	1.76	0.08

Random effects	Variance	SD			
Participants (intercept)	0.002	0.04			
Imagery	< 0.001	0.01			
Length and similarity	< 0.001	0.02			
Items (intercept)	< 0.001	0.01			
Imagery	0.004	0.06			
Residual	0.085	0.29			
AIC	509.6				
BIC	584.9				

Model: fixation percentage − imagery + (1 + imagery + length and similarity | participant) + (1 + imagery | item)

**Table 4:** Results of the mixed-effects model for fixation percentage in the relevant, blank quadrant under no cue condition offering the best fit by maximum likelihood

Fixed effects	*β*	SE (*β*)	*df*	*t*	*p*
(Intercept)	0.21	0.01	45.15	18.82	< 0.0001
Imagery	− 0.02	0.01	296.05	2.03	0.04

Random effects	Variance	SD			
Participants (intercept)	0.002	0.05			
Imagery	< 0.001	0.00			
Length and similarity	< 0.001	0.01			
Items (intercept)	< 0.001	0.02			
Imagery	< 0.001	0.02			
Residual	0.082	0.29			
AIC	473.4				
BIC	497.8				

Model: fixation percentage − imagery + (1 + imagery + length and similarity | participant) + (1 + imagery | item)

### Memory performance

See Tables 5 and 6.

**Table 5:** Results of the mixed-effects model for hit rate offering the best fit by maximum likelihood

Fixed effects	*β*	SE (*β)*	*z*	*p*
(Intercept)	1.46	0.11	13.57	< 0.0001
Length and similarity	0.24	0.05	4.86	< 0.0001
Imagery	0.18	0.04	3.96	< 0.0001

Random effects	Variance	SD		
Participants (intercept)	0.46	0.68		
Imagery	0.01	0.11		
Lexical	0.08	0.27		
Length and similarity	0.02	0.13		
Items (intercept)	0.003	0.06		
Imagery	0.002	0.05		
Lexical	0.01	0.11		
Length and similarity	0.01	0.12		
AIC	4344.1			
BIC	4490.6			

Model: hit rate − imagery + length and similarity + (1 + imagery + lexical + length and similarity | participant) + (1 + imagery + lexical + length and similarity | item)

**Table 6:** Results of the mixed-effects model for hit latency offering the best fit by maximum likelihood

Fixed effects	*β*	SE (*β*)	*df*	*t*	*p*
(Intercept)	2044.16	68.16	48.02	19.24	< 0.0001
Length and similarity	− 83.24	17.58	322.78	4.73	< 0.0001
Imagery	− 48.66	20.41	44.89	2.38	0.02
Lexical	41.49	19.51	102.85	2.13	0.04

Random effects	Variance	SD			
Participants (intercept)	208,523.2	456.64			
Imagery	5448.7	73.82			
Lexical	4291.7	65.51			
Length and similarity	778.5	27.90			
Items (intercept)	1393.9	37.33			
Imagery	2132.4	46.18			
Length and similarity	527.0	22.96			
Residual	971,565.0	985.68			
AIC	56,413				
BIC	56,541.6				

Model: hit rate − imagery + length and similarity + (1 + imagery + lexical + length and similarity | participant) + (1 + imagery + length and similarity | item)

## Footnotes

1 Imageability and concreteness are highly correlated, exhibit similar advantages in memory (see also Richardson, 1975) and thus, used interchangeably in many studies (e.g., Fliessbach, Weis, Klaver, Elger, & Weber, 2006; Nittono, Suehiro, & Hori, 2002; Reilly & Kean, 2007). Along with that, we controlled the words on concreteness in addition to imageability due to conceptual differences between them (see Vigliocco, Vinson, Druks, Barber, & Cappa, 2011 for a discussion). Concreteness is typically defined as the extent to which a word can be experienced by senses.
